# Impact of urbanization, industrialization, electrification and renewable energy on the environment in BRICS: fresh evidence from novel CS-ARDL model

**DOI:** 10.1016/j.heliyon.2022.e11457

**Published:** 2022-11-07

**Authors:** Liton Chandra Voumik, Tasnim Sultana

**Affiliations:** Department of Economics, Noakhali Science and Technology University, Bangladesh

**Keywords:** BRICS, CS-ARDL, Cross-sectional dependence, STIRPAT, Urbanization, Industrialization

## Abstract

Increased demand for water, energy, infrastructure, and other natural resources has resulted from an increase in anthropocentric activities recently, which has led to climate change, land erosion, pollution growth, and a decline in biodiversity. During the period 1972–2021, the aim of this study is how industrialization, urbanization, and renewable energy affect the environment of five industrialized economies—Brazil, Russia, India, China, and South Africa (BRICS)—implemented. Concerning slope heterogeneity, cross-sectional dependence (CSD), and a mixture of I (0) and I (1) variables, this paper used a fresh panel technique known as cross-sectionally augmented autoregressive distributive lag (CS-ARDL). When it comes to protecting water, land, and forest resources while also lowering carbon emissions, the estimate of energy, urbanization, electrification, and industrialization is favorably substantial. The findings show that rising industrialization, urbanization, income, and electrification can stimulate environmental degradation. On the other hand, renewable energy in industrialized economies may significantly lessen the environmental degradation in the BRICS region. In the BRICS region recently, urbanization is booming, all countries are expanding industrial zones, and consumption of electricity is skyrocketing. So, this research is very important in the BRICS context. The research also applied an augmented mean group (AMG) to get the impact of all variables on the environment in all BRICS countries. Because of their different economic sizes, public policy, industrial policy, population policy, immigration, and trade policy, the magnitude of impacts and signs of impacts are different. There is some evidence to suggest that renewables may be a panacea for BRICS energy security and environmental deterioration; consequently, boosting alternative energy sources, green urbanization, and environmentally-friendly urbanization should be a part of all governments' energy and environmental plans worldwide. Hence, these countries' decision-makers should re-review their population, energy, urbanization, and industrial policies to adopt a sustainable environment.

## 1 Introduction

Urbanization, industrialization, population, electricity consumption, and technology have had a significant impact on changing the economic structure. Industrialization accelerates the pace of urbanization. Rapid urbanization and industrialization create obstacles in the way of sustainable development. Environment pollution and its impact on climate change is now a topic of discussion. The negative effects of climate change due to carbon emissions are increasing. As CO2 emissions increase, the effects of climate change will become more visible because carbon emissions are associated with rising temperatures. There is a relationship with the overall context of life, including the increase in temperature, sea level rise, ice melting, drought, wildfires, and pollution. Unfortunately, global temperatures are still rising. The UN COP 26 conference suggested that action must be taken now to limit global temperatures to 1.5 °C or below by the end of this century (Dwivedi et al., 2022). The longer there is disagreement on fixing carbon emissions, the greater the risk of climate change will have to be faced. At present, several technological improvements play a vital role in environmental deterioration and decoupling development. The BRICS countries play an important role in the global population, politics, environment, and economy. The key driving forces behind the economic growth of the BRICS countries were the increased use of factors, the vast scale of the population, and abundant resources, which resulted in considerably higher economic performance than industrialized nations (Radulescu et al., 2014). Industrialized countries have a greater role to play in the world's environmental damage and negative climate change. They are leading the charge by burning more fuel, emitting large amounts of chemical waste, releasing endless mechanical and metallic nano-invisible particles into the air, and overall increasing environmental hazards. The global climate is unbalanced due to the offensive actions of these countries. BRICS countries have a great influence on world development, where growing urbanization has induced air pollution, water quality, land degradation, and environmental degradation. The effective management of urbanization in the BRICS nations will significantly affect global sustainable development (Shen et al., 2017). Industrialization is a crucial process for all nations' development and a component that encourages economic growth. But rapid industrialization also affects the quality of the environment (Dong et al., 2019). While industrialization has been shown to increase GDP in both developed and developing nations, it has had a profoundly detrimental effect on carbon dioxide emissions (Wang and Wei, 2020). Majeed et al. (2021), using the CSARDL method for short- and long-term estimates, demonstrated that urbanization and economic expansion raise carbon emissions while an abundance of natural resources, the use of renewable energy, and economic globalization lower emissions of carbon dioxide. Environmental-related technology (ERT) has a significantly negative impact on environmental degradation and suggests that the BRICS economies increase investments in eco-friendly technologies to attain a supportable environment in the future (Hussain and Dogan, 2021). According to estimates from CS-ARDL, CCEMG, and AMG, the ASEAN-6 economies' environmental sustainability is being harmed by increased use of transportation infrastructure, increased urban migration, economic growth, and the increase of environmental-related taxes thought to be beneficial for environmental protection (Huang et al., 2021a, Huang et al., 2021b). Li and Lin (2015) applied dynamic panel threshold models and showed carbon emissions are exerted differently by industrialization and urbanization at various levels of GDP per capita. Zhang (2021), recommended that economic growth significantly enhances carbon emissions in BRICS countries, but the emissions levels are different for every single BRICS country at their development stages. Using the CS-ARDL method, the empirical study done by Azam et al. (2022) showed that energy consumption, urbanization, industrial growth, and GDP per capita income all contribute to carbon emissions. They advised SAARC nations to invest in renewable energy sources and carefully plan their urbanization to lower carbon emissions. Development cannot be sustainable by destroying the natural environment. By using eco-friendly technology with good planning, it is feasible to carry out development operations without harming the environment. Renewable energy has gained wide acceptance in the world today. Most countries have set targets for renewable energy use to meet their electricity and energy needs. It requires the application of the appropriate industrial, urban, and energy policies.

BRICS countries are among the leading countries in terms of GDP size, technology, globalization index, and renewable energy. However, BRICS members are the largest emitters, which are responsible for a quarter of the world's CO2 emissions (Sharma et al., 2021). At present, combating climate change and, therefore, reducing greenhouse gas emissions is an important priority on the agenda of BRICS countries. The primary objective of this paper was to analyze how various key variables, including population, income, urbanization, industrialization, renewable energy consumption, and electricity consumption, relate to environmental degradation in the BRICS region. Due to unplanned urbanization, rapid industrialization, indiscriminate use of natural resources, producing electricity from fossil fuels, and lack of environmental awareness, biodiversity is being endangered in various urban, rural, and regional areas in the BRICS region. BRICS countries are trying to be world economic superpowers. These countries are increasing rapid economic growth, unplanned development, and energy consumption without considering sustainable environmental conditions. Based on these premises, the paper adds to the existing literature in the following ways: first, the study uses data from 1972 to 2021 to investigate the effect of population, income, urbanization, industrialization, renewable energy sources, and electricity consumption on environmental degradation in the BRICS. Secondly, the BRICS zone is highly interconnected with trade, bilateral cooperation, and cultural exchange. Cross-sectional dependence problems and slope heterogeneity problems are present in the data. The paper applied second-generation unit root tests, cointegration tests, and newly developed cross-sectional autoregressive distributive lag (CS-ARDL) to handle the problems of data. In the theoretical context, the paper applied STIRPAT (Stochastic Impacts by Regression on Population, Wealth, and Technology) model to figure out how population, wealth, and technology are related to environmental degradation. To fulfill our secondary objective, the paper applied the AMG estimator to identify specific environmental impacts in every BRICS country. Each country needs to have its own information because BRICS countries are different in population, GDP, renewable energy consumption, industrialization, urbanization, and CO2 emissions. Finally, the research suggests how policymakers will make policies considering industrialization, urbanization, population, and electricity consumption.

## 2 Literature review

Wang et al. (2016) employed a Granger causality framework to look at the association between urbanization and carbon emissions in the BRICS nations from 1985 to 2014. They employed time series methods such as panel unit root, co-integration, and causality tests that took into account cross-sectional dependency, non-stationaries, and heterogeneities to calculate the long-term causal influence of urbanization on carbon emissions. Between 2001 and 2014, Rehman and Rehman (2022) studied the effect of population increase, urbanization, economic development, and energy consumption on carbon emissions in the five most populous Asian nations. The rapid growth in the population and a thriving economy have made India a major contributor to global warming, as shown by their analysis of the country's carbon footprint. even though urbanization and energy use are major factors in China and Pakistan's emissions. Using the Durbin-Hausman and Westerlund panel co-integration tests for the BRICS countries, Erdogan (2021) found a long-term relationship between carbon emissions and technological advancement. As a result of his research using Dynamic Common Correlated Effects, he recommended that they put money into R&D to reduce carbon emissions. Environment-related technology and institutional quality were examined in a study (Hussain and Dogan, 2021) by employing CSD (cross-sectional dependence) statistics, panel unit root tests, Pedroni, Kao, and Wester Lund cointegration, cross-sectional augmented autoregressive distributive lag (CS-ARDL), common correlated effects mean group (C.C.E.M.G), and augmented mean group (A.M.G). Their research led them to the conclusion that economic activity causes an increase in pollution and that the EKC hypothesis does not hold for the BRICS countries. Using a dynamic panel data set of 73 countries from 1971 to 2010, Li and Lin (2015) analyzed the effects of urbanization and industrialization on carbon emissions and how they vary across economic development levels. Using the STIRPAT setting, this research re-estimates the connection using multiple panel data models that account for heterogeneity and the “ratchet effect”. Using the iPETS (integrated Population-Economy-Technology-Science) model, O'Neill et al. (2012) assessed the effects of a realistic range of urbanization mechanisms on energy consumption and carbon emissions in China and India. They concluded that urbanization shifts have an impact on total emissions and energy use that is a little out of proportion. According to empirical findings, this effect is an effect of economic growth brought on by rapid urbanization. Non-linear iterative partial least squares (NIPALS) were used in a study by Asumadu-Sarkodie and Owusu (2017) on carbon dioxide emissions in Senegal, and the results showed that industrialization and electricity use had a positive correlation with CO_2_ output, while economic growth and urbanization had a negative one. Carbon dioxide emissions, renewable energy consumption, energy intensity, economic growth, industrialization, and urbanization in China were analyzed by Liu and Bae (2018) using the VECM and ARDL. Based on empirical evidence, it seems that rising rates of real GDP, industrialization, urbanization, and energy intensity are all associated with rising rates of carbon dioxide emissions at the rates of 0.6%, 0.3%, 1.0%, and 1.1%, respectively. Between 1990 and 2015, Wang and Su (2019) used the Granger causality test, Johansen co-integration theory, and the Tapio model to examine how industrialization and urbanization affected China's decoupling of economic growth from carbon emissions. The results show that GDP and energy structure positively affect DE, while industrialization, urbanization, and technological progress negatively affect DE over the long term. Salahuddin et al. (2019) analyzed the effects of economic development, urbanization, and globalization on carbon emissions in South Africa using time-series annual data from 1980 to 2017. Although only long-term substantial emissions effects of globalization were identified, this empirical study using the ARDL cointegration test found that urbanization increased CO_2_ emissions both in the short and long term. From 1995 to 2018, researchers Huang et al., 2021a, Huang et al., 2021b tracked the ASEAN economies' growth, urbanization, transportation, and greenhouse gas emissions. The greenhouse gas emission impacts were evaluated using the novel CS-ARDL model, with CS-ARDL estimations supported by both the A.M.G and the C.C.E.M.G. The empirical results showed that CO_2_ emissions increase with urbanization, transportation, and economic growth but are reduced by environmentally related taxation. Carbon emissions from the BRICS economies can be reduced thanks to the use of renewable energy, as analyzed by Leitão et al. (2021). Income, renewable energy, environmentally friendly technology, green growth, environmental taxes, and monetary development all affect carbon dioxide emissions, according to a study conducted in a few ASEAN countries by Saleem et al. (2022) using CS-ARDL. Industrialization and economic growth, as predicted by the ARDL elasticity coefficients, are found to have a negative relationship with environmental degradation and to decrease carbon emissions, as stated by Elfaki et al. (2022). Using the CS-ARDL method, Safi et al. (2021) found that environmental taxes and R&D will significantly reduce carbon emissions and help the G-7 countries reach their goal of carbon neutrality.

According to the aforementioned literature, various studies have produced conflicting findings regarding the causes of environmental deterioration. In this study, the paper applies fresh evidence from the novel CS-ARDL model, which has been absent in the majority of earlier investigations. CS-ARDL is a method that has several benefits, such as controlling for cross-sectional dependence, endogeneity, serial correlation, integrating features of the variables, and making short-term and long-term projections (Usman et al., 2022). The majority of the research work described above demonstrates that many investigations have produced inconsistent findings regarding the causes of environmental degradation. The variables and models gathered from different countries may be the causes of the contradicting results because they did not capture all the relevant factors. There is a gap in that research work: very few studies have looked at what causes the environment to get worse in the BRICS countries, and the STIRPAT hypothesis has not been taken into account in the analytical framework. So, the paper considers the STIRPAT model to figure out how population, wealth, and technology affect environmental degradation. To stop the environment from getting worse in BRICS countries, the policies that are already in place need to be replaced with new ones.

## 3 Methodology

### 3.1 Data

The variable lists for this paper are presented in Tables 1 and 2 provides a summary of the descriptive statistics about each variable. Data for all indicators were gathered from the World Development Indicator (WDI). We've included both descriptive statistics and a full list of variables for your perusal and analysis.

**Table 1:** Variables’ name and details.

Variable Name	Log Form	Indicators' name
CO_2_ emissions	L (CO_2_)	CO_2_ emissions (kt)
Population	L (POP)	Total population
GDP	L (GDP)	GDP (constant 2015 US$)
Renewable energy	L (REN)	Use of renewable sources of energy (as a percentage of total consumption)
Urbanization	L (URBA)	Urban population (% of the total population)
Electricity	L (ELEC)	Access to electricity (% of the population)
Industrialization	L (INDUS)	Industry (including construction), value added (% of GDP)

**Table 2:** Descriptive statistics.

VARIABLES	N	mean	sd	min	max
LCO_2_	245	13.83	7.111	12.20	16.20
LGDP	228	24.97	7.839	25.52	30.31
LPOP	245	18.95	2.999	16.96	21.07
LREN	190	1.682	1.616	1.157	4.072
LURBA	220	18.24	2.811	12.23	20.58
LELEC	215	5.641	3.124	1.254	8.808
LINDUS	218	3.163	1.015	1.024	3.872

### 3.2 Theoretical background and model construction

To achieve the goal of this paper we need a model related to the impact of urbanization, industrialization, electricity consumption, and renewable energy on environmental degradation. Our dependent and independent variables suggest applying the famous STIRPAT model. Which stands for stochastic (ST) impacts (I) through regression (R) on population (P), affluence (A), and technology (T), was developed by Dietz and Rosa (1997) as an improved version of IPAT. The four main variables in our paper are impact, population, affluence, and technologies, which are the main component of the STIRPAT model.

In 1970, Holdren et al. (2012) established the now-commonly used IPAT identification approach, which demonstrates that population size and growth rate have been significant contributing factors to environmental damage. The well-known IPAT equation was used to figure out that affluence, technology, and population were the three main determinants of environmental change (York et al., 2003). Huang et al., 2021a, Huang et al., 2021b used the IPAT model to learn more about how human activities damage the environment and how to stop it. The IPAT model doesn't take into account that the effects of the main environmental factors aren't always the same, they improved the STIRPAT model by coming up with the idea of ecological elasticity (York et al., 2003). STIRPAT overcame IPAT's shortcomings and was applied to empirically test the premise in Eq. (1).

The basic STIRPAT model follows the following form:

(1) $$Ii=C.Pi^{\alpha }.Ai^{\beta }.Ti^{\gamma }.\varepsilon i$$

Natural logarithmic was taken on both sides to convert the equation to a log-linear form in Eq. (2):

(2) lnIi=C+αlnPi+βlnAi+γlnTi+εi

Here, C means the intercept term, a random error term (ε), and α, β, and γ are the estimated exponents of the population (P), affluence or resources (A), and technological innovation or improvement (T) in the STIRPAT model. The STIRPAT models analyzed the interrelationships between technological elements like trade liberalization, energy consumption, human capital, industrialization, urbanization, nuclear power, technological innovation, and financial developments (Huang et al., 2021a, Huang et al., 2021b). Bargaoui et al. (2014) utilized the STIRPAT model to examine the Kyoto Protocol's pledges on carbon dioxide emissions and the factors that contribute to environmental degradation. According to Hussain et al. (2022), in a study done in the BRICS economies, consistent economic policies are needed to fully realize the benefits of environmental technologies. This study tested the effects of economic policy uncertainty on ecological footprints using the STIRPAT model, as well as the energy structure and investment in environmental technologies. The main objective of our study is to reveal the impact of population, GDP, urbanization, industrialization, renewable energy, and electricity on environmental degradation. That's why the research uses the STIRPAT model to figure out how population, wealth, and technology affect environmental degradation. Thus, the model takes the following form in Eq. (3):

(3) $$LCO_{2}=\alpha_{O}+\alpha_{1}LPOP_{it}+\alpha_{2}LGDP_{it}+\alpha_{3}LREN_{it}+\alpha_{4}LURBA_{it}+\alpha_{5}LELEC_{it}+\alpha_{6}LINDUS_{it}+\varepsilon_{it}$$

where CO_2_ represents the carbon emissions, which are determined by population (LPOP), gross domestic product (LGDP), urbanization (LURBA), industrialization (LINDUS), renewable energy (LREN), and electricity (LELEC) and a random error term (ε). Here, countries and years are shown for i and t.

When assessing environmental health, carbon emissions are a useful yardstick. An application of the STIRPAT model in China between 1980 and 2010 yielded empirical evidence that demographic and economic characteristics, as well as the country's degree of urbanization and industrialization, all play a role in the country's rate of carbon dioxide (CO_2_) emissions growth. CO2 emissions, on the other hand, can be lowered thanks to technological progress, shifting patterns of energy consumption, and international commerce (Wang et al., 2013). Applying the STIRPAT model, Bai et al. (2019) analyzed the economic and demographic development of sixty-four different Chinese cities. They analyzed whether urbanization increased carbon emissions while GDP growth decreased them. To accurately assess the impact of urbanization on CO_2_ emissions, particularly the technology-related factor, Zhang and Zhao (2019) substituted other variables like energy utilization, energy intensity, energy sanitation, service level, and research and development funds into this STIRPAT model place of technology.

### 3.3 Econometric methodology

Firstly, our works are based on cross-sections where countries are interdepended with each other. On the other hand, the number of T is larger than the number of N, we are using panel cointegration analysis in this investigation. Here, we use cross-sections spanning 50 years and five different countries. Traditional methods, such as fixed and random effect models, are more appropriate if N > T, but we cannot use them if T > N (Zoundi, 2017). The five countries in this research are highly linked by trade, contract, cultural exchange, and other perspectives. Also, these countries are rising economies and world economic leaders after G7 (Group of Seven). So, based on the panel data nature, diagnostics, and findings these five cross-sections have cross-sectional dependence, slope heterogeneity, and mixed order (I (0) and I (1)) stationary problem. Because of mutual collaboration and cooperation in the BRICS region, the paper applies the CSD test. Though all BRICS countries are economic superpowers the magnitudes of indicators are different. So, the paper applies the slope homogeneity test. After confirming CSD and SH problem this work must apply the second-generation unit root test and cointegration test. CIPS and CADF (Pesaran, 2007) tests are applied to managing CSD and SH problems. Once the unit root has been tested the paper's next move is to cointegration test. The paper applied the second-generation cointegration test (Westerlund, 2007). The Westerlund (2007) test incorporates CSD, heterogenous effects, and nonstationary problems in the data. Table 7 presents cointegration results. After considering all of these required tests the paper applied the CS-ARDL method. Because of common energy, trade, and other cross-sectional shocks, global financial crisis, and globalization the data facing CSD problem. So, the CS-ARDL technique can handle CSD, endogeneity, and SH problem (Hussain et al., 2020; Zaidi et al., 2021; Khan et al., 2020).

The outcome of CS-ARDL shows in Table 8. After applying second-generation CS-ARDL the next step is to check robustness. In the presence of CSD and SH, the traditional approaches may provide spurious, biased, and partial estimates. The AMG estimator is applied to check the effect on each country. These estimators are also well-suited to manage heterogeneity and cross-sectional dependence.

**Figure 1:**
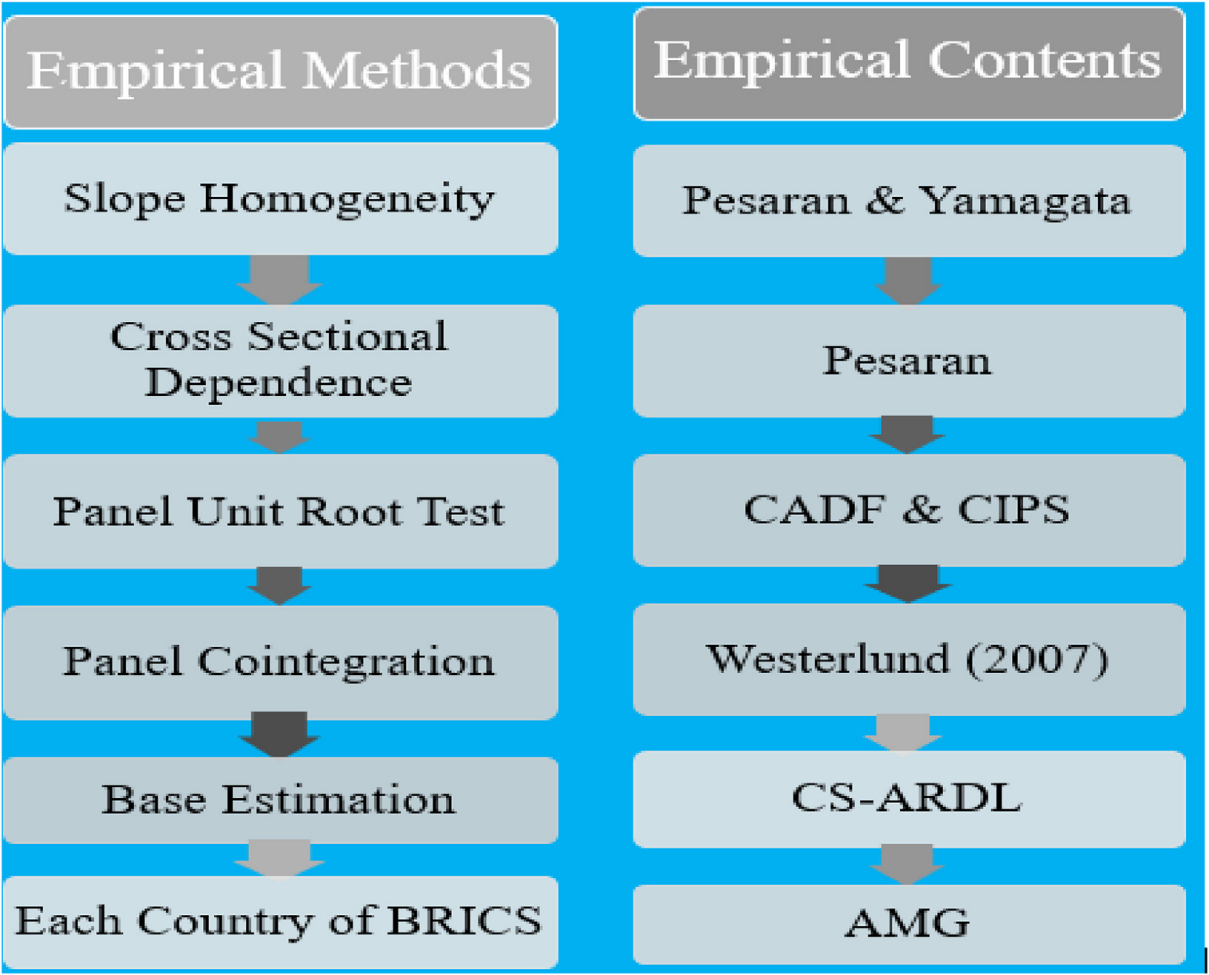
The framework of the CS-ARDL process.

The empirical steps of this paper are organized and serially written in Figure 1.

#### 3.3.1 Slope homogeneity test

In panel data econometrics weighted of all countries are different so heterogeneity in slopes is crucial. Initial slope heterogeneity is analyzed using the test developed by Pesaran and Yamagata (2008). This test is based on how all countries’ weighted slope is spread out. The following Eq. (4) provides the corresponding test statistics:

(4) $$\overset{\text{ˇ}}{\Delta }=\sqrt{N}\left(\frac{N^{-1}S\%-k}{\sqrt{2k}}\right)\;and\;{\overset{\text{ˇ}}{\Delta }}_{adj}=\sqrt{N}\left(\frac{N^{-1}S\%-k}{\sqrt{\frac{2k\left(T-k-1\right)}{T+1}}}\right)$$

#### 3.3.2 Cross-section dependence test

As a result of globalization, increased economic integration, and decreased trade barriers, cross-section dependence is increasingly prevalent in panel data econometrics (Tufail et al., 2022). Findings that are skewed, inconsistent, and misleading can be the result of failing to account for cross-section dependence and instead presuming that cross-sections are independent of one another (Westerlund and Edgerton, 2007). The authors of this work use a test for weakly exogenous cross-section dependency (Pesaran, 2015) in large panel data econometrics to determine whether or not there is cross-section dependence. This is done so that they can identify whether or not there is cross-section dependence. The following Eq. (5) is a summary of the standard equation used in C.S.D. tests.

(5) $$CSD=\sqrt{\frac{2T}{N\left(N-1\right)N}\left(\sum_{i=1}^{N-1}\sum_{K=i+1}^{N}{\hat{Corr}}_{i,t}\right)}$$

#### 3.3.3 Unit root test

Harris and Tzavalis, ADF, Philips Perron, Breitung, Maddala, and Hadri these famous first-generation unit root test will not work when CSD and SH problem is present. Thus, a CIPS and CADF (Pesaran, 2007) second-generation unit root test was used to investigate the stationarity of the variables despite the presence of CSD and slope heterogeneity. Eq. (6) shows how calculating a cross-sectional mean of ti is necessary for a CIPS calculation.

(6) $$CIPS=\frac{1}{N}\sum_{i=1}^{N}t_{i}\left(N,T\right)$$

Thanks to its effectiveness in dealing with CSD and heterogeneity, CIPS is gaining popularity in the academic world. The unit-root series is the baseline hypothesis. If the variable is at first difference stationarity, the test also suggests running a cointegration test before moving on to parameter estimation.

The CADF method is used to obtain the statistics used by CIPS. Conversely, Eq. (7) for CADF (Cross-Augmented Dicky Fuller) is as follows:

(7) $$\Delta Y_{it}=\varphi_{i}+\zeta_{i}Y_{i,t-1}+\delta_{i}{\bar{Y}}_{t-1}+\sum_{j=0}^{P}\delta_{ij}{\bar{Y}}_{t-1}+\sum_{j=1}^{P}\lambda_{ij}{\Delta Y}_{i,t-1}+\varepsilon_{it}$$

where ${\bar{Y}}_{t-1}$ and $\Delta Y_{i,t-1}$ are average for lagged and first difference of each cross-sectional series.

#### 3.3.4 Co-integration testing

A heterogeneous estimating strategy is required for co-integration detection because of the existence of CSD, heterogeneity, and non-stationarity in the data (Larsson et al., 2001; Pedroni, 2004; Kao et al., 1999; Pedroni, 2001). The method developed by Westerlund (2007) accounts for variation in the slope, coefficient of determination, and correlated errors. This method reliably forecasts cointegration properties in cross-sectionally dependent heterogeneous panel data. It also computes error-corrected statistics for testing for no cointegration across four panels. The typical form of this second-generation Westerlund (2007) cointegration test consists of the following four Eqs. (8), (9), (10), and (11):

(8) $$G_{\alpha }=\frac{1}{n}\sum_{i=1}^{N}\frac{\alpha_{i}^{\prime }}{SE\left(\alpha_{i}^{\prime }\right)}$$

(9) $$G_{t}=\frac{1}{n}\sum_{i=1}^{N}\frac{T\alpha_{i}^{\prime }}{\alpha_{i}^{\prime }\left(1\right)}$$

(10) $$P_{t}=\frac{\overset{´}{\alpha }\;}{SE\left(\overset{´}{\alpha }\;\right)}$$

(11) $$P_{\alpha }=T\alpha^{\prime }$$

There are a few different types of group means statistics (Gt and Ga), in addition to panel means statistics (Pt and Pa), and each of these types has its own set of abbreviations. When the model variables are assumed to be unrelated, or “null”, and the alternative hypothesis is “there are cointegrating relationships”, then the corresponding test statistics are expected.

#### 3.3.5 CS-ARDL test

After confirming the presence of a long-run link using Westerlund's (2007) Panel Cointegration test, the current investigation will employ a recently developed technique: the cross-sectionally augmented autoregressive distributed lags model (CS-ARDL). In this research, both long-term and short-term assessments are conducted using the CS-ARDL assessment created by Chudik and Pesaran (2016). In comparison to the mean group (MG), pooled mean group (PMG), common correlated effect mean group (CCEMG), and augmented mean group (AMG), this test requires far less time and effort (Wang et al., 2021). By another definition, this method solves seemingly undiscovered issues of endogeneity, non-stationarity, mixed-order integration, SH, and CSD. This is because getting inaccurate estimation results is directly related to ignoring unobserved common components. The CS-ARDL can be represented by the following Eq. (12).

The equation for the model is expressed as:

(12) $$LCO2=\alpha_{it}+\sum_{j=1}^{P}\beta_{it}LCO2_{i,t-j}+\sum_{j=0}^{P}\gamma_{it}X_{t-j}+\sum_{j=0}^{3}\delta {\bar{Y}}_{t-j}+\varepsilon_{it}$$

where $\bar{Y_{t}}={\left(\Delta L\bar{CO2_{t}},{\bar{X_{t}}}^{\prime }\right)}^{\prime }$ and

$$X_{it}={\left({LPOP}_{it}LGDPpc_{it}{LREN}_{it}{LURBA}_{it}L{ELEC}_{it}L{INDUS}_{it}\right)}^{\prime }$$

## 4 Results and findings

The outcomes of the slope homogeneity test conducted by Pesaran and Yamagata (2008) are shown in Table 3. The findings show that the model's coefficients are non-uniform and that the slope varies among countries.

**Table 3:** Slope heterogeneity test.

Slope homogeneity tests Δ statistic P-value		
$\overset{ˇ}{\Delta }$ test	20.403∗∗∗	0.000
${\overset{ˇ}{\Delta }}_{adj}$ test	22.262∗∗∗	0.000

For the test of slope heterogeneity, the assumption that slope coefficients are all the same serves as the null hypothesis. The level represented by ∗∗∗ is lower than 1%.

Cross-sectional dependence testing is a required first step in any econometric analysis of panel data. Tabulated in Table 4 is the outcome of a CSD test for weak cross-sectional dependence by Pesaran (2015). It demonstrates that panel data are subject to cross-sectional dependence. In other words, similar economic and political circumstances mean that LCO_2_, LPOP, LGDP, LREN, LURBA, LELEC, and LINDUS all have cross-sectional dependence.

**Table 4:** Results of cross-sectional dependence analysis.

Variable	Test statistics (P-value)
LCO_2_	22.35∗∗∗ (0.00)
LPOP	18.91∗∗∗ (0.00)
LGDP	23.32∗∗∗ (0.00)
LREN	22.19∗∗∗ (0.00)
LURBA	19.26∗∗∗ (0.00)
LELEC	18.09∗∗∗(0.00)
LINDUS	12.33∗∗ (0.047)

Note that the levels of significance at 10%, 5%, and 1% are explained by the symbols ∗, ∗∗, and ∗∗∗, respectively, while the values that are enclosed in parenthesis contain the P-values.

The next phase of this work involves verifying the correct sequence of operations when integrating multiple data sets. Tables 5 and 6 show the results of applying the CIPS and CADF unit root tests for this purpose. All variables are found to be integrated at the level and first difference. Consequently, LCO_2_, LPOP, LGDP, LREN, LURBA, LELEC, and LINDUS are all trending in the same direction. As a means of achieving this goal, we have implemented second-generation unit root tests. The results of these unit root tests are shown in Tables 5 and 6, which suggest that certain variables are stationary at the level or I (0), while others become stationary after differencing or I (1). In conclusion, we are in a position to assert that each of the variables that were investigated may be placed into one of two distinct categories: I (0) or I (1), but not I (2).

**Table 5:** CIPS unit root test.

Variable	CIPS test	
	At Level	1st differences
LCO_2_	−1.957	−3.211∗∗∗
LPOP	−2.260∗∗	
LGDP	−2.249∗∗	
LREN	−2.269∗∗∗	
LURBA	−1.171	−6.190∗∗∗
LELEC	−3.551∗∗∗	
LINDUS	−0.920	−3.254∗∗∗

Note that the levels of significance at 10%, 5%, and 1% are explained by the symbols ∗, ∗∗, and ∗∗∗, respectively, while the values that are enclosed in parenthesis contain the P-values.

**Table 6:** CADF unit root test.

VariableCADF test						
	At Level			1st differences		
	T-bar	Z-t-tilde-bar	P value	T-bar	Z-t-tilde-bar	P value
L**CO**_**2**_	−3.254	−1.642	0.047			
**LPOP**	−2.6.6	−1.966	0.025			
**LGDP**	−2.041	−0.621	0.267	−3.051	−3.023	0.001
**LREN**	−2.333	−1.320	0.093			
**LURBA**	−1.173	1.444	0.926	−4.745	−7.053	0.000
**LELEC**	−1.458	0.765	0.778	−4.795	−7.173	0.000
**LINDUS**	−1.794	−0.034	0.486	−4.983	−7.620	0.000

Note that the levels of significance at 10%, 5%, and 1% are explained by the symbols ∗, ∗∗, and ∗∗∗, respectively, while the values that are enclosed in parenthesis contain the P-values.

**Table 7:** Cointegration tests.

Variable	Westerlund test for cointegration		
	Value	Z-value	P-value
Gt	−4.17	1.568	0.00
Ga	−7.52	3.964	0.99
Pt	−8.25	2.617	0.00
Pa	−3.47	3.007	1.00

**Table 8:** Outcomes of CS-ARDL.

Variables	Long run results		Short run results	
	Coefficients	Std. Err.	Coefficients	Std. Err.
**LPOP**	−.0095	3.022	−.0085	1.402
**LGDP**	.0425∗	.0727	.0422	.0524
**LREN**	−.4299∗∗∗	.1395	−.3295∗∗∗	.893
**LURBA**	1.1830∗∗∗	.5467	.942∗∗∗	.2482
**LELEC**	.1322	.0395	.1021	.0823
**LINDUS**	.1732∗∗∗	.0807	.1520	.0462

The subject of whether or not the variables are long-run cointegrated may be addressed once the unit root tests have been looked at. Here, we use the cointegration tests developed by Westerlund (2007), and the results are shown in Table 7. At the 1% level of significance, neither the Gt nor the Pt data in Table 7 are consistent with the Westerlund null hypothesis (2007). Both of our panel cointegration experiments show that environmental deterioration, population, income, renewable energy, urbanization, industrialization, and electrification are all interconnected in a long-run equilibrium connection.

Table 8 displays the outcomes of the CS-ARDL analysis. The discussion will begin with an examination of the outcomes in the long term. Depressing as it may seem, the anticipated long-run coefficient of the population has a relatively modest impact on total carbon emissions. This indicates that a 1% increase in BRICS population growth would result in a 0.0095% decrease in carbon emissions. The findings demonstrate how the BRICS countries' use of renewable energy sources has reduced their environmental footprint. To be more precise, for every 1% increase in the usage of renewable energy, carbon emissions are reduced by 0.4299%. We present GDP coefficient estimates that are both positive and statistically significant. In such a case, it may be concluded that GDP is related to carbon emissions. For every percentage point GDP grows, emissions go up by 0.042%. Urbanization, electricity, and industrialization are positively connected with long-term CO2 emissions. Carbon emissions rise by 1.183 percent for every one percentage point of LURBA urban population growth. Rapid urbanization is a barrier to sustainable development. Similarly, Suki et al. (2021) found a favorable and statistically significant connection between urbanization and ecology. Several other researchers have corroborated these findings, and they believe that by limiting urbanization, the purpose of maintaining a sustainable environment can be attained (Bai et al., 2019). However, greater power use typically spurs economic expansion and, hence, environmental damage. According to the estimations of LELEC, a rise of 1% in global power consumption results in an increase of 0.1322% in carbon emissions. Last but not least, there is positive significance in the connection that industrialization has with carbon emissions. In particular, a 1% increase in industrial activity is associated with a 0.1732% increase in carbon emissions, suggesting that it can hasten environmental degradation. In the short term, both population and the use of renewable energy can be seen to have a mitigating effect on carbon emissions. Carbon emissions are reduced by 0.0085% and 0.3295 percent for every one percent increase in both population and renewable energy, respectively. While population growth has little effect, renewable energy has substantial effects. Income, urbanization, electricity, and industrialization have a severe impact on environmental deterioration in the short run also. A 1% rise in wealth, urbanization, electrification, and industrialization proportionately raises the carbon emissions by 0.0422%, 0.942%, 0.1021%, and 0.1520%, respectively. The outcomes obtained in the short run demonstrate that LURBA has a considerable influence but LGDP, LELEC, and LINDUS have insignificant impacts on carbon emissions.

The aforementioned studies show that when combined with CS-ARDL, AMG provides high levels of reliability (Ahmed et al., 2022). The authors used the AMG estimator to calculate the long-run elasticities for all BRICS nations. Table 9 displays the outcomes of the AMG estimator's testing procedures. Table 9 shows that, except for Russia, the BRICS nations have all found the consequences of renewable energy to be negative and considerable. This suggests that a rise in renewable energy consumption in Brazil, India, China, and South Africa will lead to a decline in those countries' CO2 emissions. Renewable energy in India has the potential to cut carbon dioxide emissions more than in any other country. However, the population impact on the environment is negative for every country except South Africa. There are many degrees of significance for each of the coefficients. No significant correlations between BRICS nations' per capita true GDP and environmental effect were found. Furthermore, rising urbanization in Russia has been linked to a decline in the country's CO2 emissions. However, in China and South Africa, rising urbanization is accompanied by rising CO2 emissions. Brazil and India have relatively small urbanization coefficients. Russia has had the most negative effect (7.057) from urbanization on CO2 emissions. In all BRICS nations, rising power consumption is accompanied by higher levels of carbon dioxide emissions. This is because fossil fuels are widely used to generate power in BRICS nations. In Russia, the effect of energy usage on CO2 emissions was found to be the most significant (0.758). The effects of industrialization on Brazil, China, and South Africa have been substantial and beneficial. Other nations, including Russia and India, were not found to have any major effects since the investigators could not find any correlations between industrialization and CO2 emissions. Their economies have different sizes, hence the magnitude and sign of the effects are varied.

**Table 9:** AMG estimation results.

Country	LGDP	LPOP	LRENEW	LURBA	LELEC	LINDUS
Brazil	−.120 (.124)	6.055∗ (4.503)	−.454∗∗∗ (.098)	−1.908 (2.601)	.297∗∗∗ (.100)	.070∗ (.024)
Russia	.178 (.142)	2.537∗∗ (8.205)	−.051 (.065)	−7.057∗∗∗ (7.095)	.785∗∗∗(.250)	.034 (.071)
India	.220 (.169)	3.273∗ (2.251)	−.823∗∗∗ (.251)	−3.214 (3.144)	.208∗∗∗ (.101)	−.034 (.144)
China	.101 (.173)	6.461∗∗∗ (2.607)	−.546∗∗∗ (.112)	2.655∗∗∗ (.842)	.406∗∗∗ (.133)	.022∗∗ (.257)
South Africa	.060 (.432)	−6.154∗∗ (8.346)	−.2354∗∗∗ (.0797)	9.017∗∗ (8.006)	.356∗∗ (.180)	.611∗∗ (.254)

## 5 Discussion

According to Table 8, population growth has a negligible effect on environmental deterioration and the standard of air, land, forests, and water in the BRICS countries. Usman et al. (2022) earlier research corroborating this finding indicated that people in developed economies tend to have higher levels of education and a greater concern for the environment. Eco-friendly technology and renewable energy sources are widely used in developed economies. Renewable energy has been shown to enhance environmental quality and contribute to a more favorable ecosystem. Hydroelectric, wind, biomass, tidal, and solar power are all examples of renewable energy sources that can be used without negatively impacting the environment. Liu et al. (2020), Mehmood (2021), Mehmood (2022a), Voumik et al. (2022a), and Mehmood (2022b) all discovered a comparable effect in renewable energy and carbon emissions, and all four pieces of the prior study concluded that renewable energy can reduce CO_2_ emissions. The use of renewable energy has been shown to reduce carbon emissions in OECD countries (Ahmed et al., 2022; Tian and Li, 2022). Growth in the economy harms the environment since it uses up scarce resources like water, land, and forests. Income growth is associated with increased production, demand for fossil fuels, human requirements, and resource use, all of which degrade environmental quality. Positive and important findings have been defended by many other researchers. Usman et al. (2022) showed that a 1% increase in GDP raises the environmental footprint by 0.56% in industrialized economies is compelling. Those studies by Ahmed et al. (2020), Tufail et al. (2022), and Liu et al. (2020) also found a correlation between rising wealth and deteriorating ecosystems. Even though it has been established that the generation of electricity from fossil fuels harms the environment, the positive coefficients may be explained by the fact that the BRICS nations utilize more electricity to spur economic development. This finding is consistent with those of Shahbaz et al. (2014), Voumik et al. (2022b), Voumik et al. (2022c), and Lean and Smyth (2010). The impact of industrialization findings has been supported by Liu and Bae (2018). Through regulating industrialization, the goal of establishing a sustainable ecosystem can be attained. Empirically, our results disagree with those of Li and Lin (2015), Elfaki et al. (2022), and Sharma (2011), all of whom indicate that industrialization has a major and detrimental effect on environmental degradation in many nations. In Table 9, the impacts of population, urbanization, industrialization, and electricity consumption on CO2 emissions are different. The impact of population growth on the environment is positive except in South Africa. Renewable energy can reduce CO2 emissions for all countries except Russia. Population, electricity consumption, and urbanization can all increase CO2 emissions in China. Only renewable energy can reduce environmental pollution.

## 6 Conclusion

The primary objective of this paper was to analyze how various key variables, including population, income, urbanization, industrialization, renewable energy consumption, and electricity consumption, relate to environmental degradation in the BRICS region. Population, renewable energy, urbanization, electricity use, and industrialization are all factors we consider in this study of the STIRPAT model's relationship between human activity and atmospheric carbon dioxide levels. To validate the proposed nexus, several statistical techniques, such as the SH, CSD test, second-generation CIPS and CADF unit roots tests, second-generation co-integration (Westerlund, 2007) test, and the CS-ARDL are utilized. These techniques are applied to the period that spans from 1972 to 2021. Because BRICS countries are interconnected through trade, tourism, and international agreements, the CSD problem is present in the data. The CS-ARDL findings indicate that the use of renewable sources of energy contributes to a reduction in the rate of environmental degradation in the BRICS countries. It has been discovered that population, GDP, urbanization, industrialization, and income are all factors that contribute to negative effects on the environment. The research also applied the AMG estimator to find out the impact on each country's environment. In the AMG estimation, findings show that renewable energy can minimize CO2 emissions for every BRICS country, and except for Russia, all coefficients are significant. The research found that electricity consumption increases CO2 emissions in all BRICS countries, and the coefficients are significant. For the BRICS economies, urbanization has had mixed results. The impact of GDP is positive except in South Africa, where coefficients are insignificant.

## 7 Policy recommendation

Based on the empirical findings, some significant policy implications can be suggested.

• All BRICS countries' populations have a detrimental impact on the environment, except South Africa. Both India and China have massive population explosions. Brazil's and China's massive populations have serious consequences for their environments. It is recommended that the BRICS countries adopt two different demographic policies. Making everyone aware of the need to protect the environment is the first step. In this way, the local community may continue shouldering its share of the burden of maintaining a healthy ecosystem. The second goal is to slow the rate of expansion of the human population and shift the focus from sheer numbers to quality of life.
• Renewable energy is the only factor that all BRICS countries can agree on to slow down environmental degradation (only the Russian coefficient is insignificant). The BRICS countries may decide to increase their spending on renewable energy production and processing to take advantage of the benefits. Providing financial incentives to companies that transition to renewable energy sources would promote sustainable development over the long term. Solar, wind, geothermal, biomass, hydro, and tidal wave energy are all readily available in large quantities in all BRICS countries. With the appropriate application of these resources, the BRICS can achieve their goal of sustainable development. Therefore, for a long-term sustainable environment, all BRICS countries should adopt renewable energy.
• This article argues that governments should pay more attention to sustainable urbanization to protect environmental quality by implementing effective policies and strategies because urbanization significantly increases CO2 emissions in China and South Africa but decreases CO2 emissions in Russia and India. Both public and private entities in China and South Africa should work toward decentralizing their operations away from the cities and promoting sustainable forms of urbanization. The mayors of major cities in China and South Africa need to take the necessary measures to improve their respective transportation networks by making them more energy efficient. By soaking up greenhouse gases, urban forests can help lessen the effects of air pollution.
• All of the BRICS nations have strong correlations between their electricity usage and environmental deterioration. In Russia, the consumption of electricity has the most significant influence on the deterioration of the natural environment. Coal, petroleum, and natural gas are the primary sources of BRICS's electricity. So, BRICS nations should use renewable electricity generation sources to protect the environment. The replacement of fuels, the incorporation of nuclear and renewable energy sources into the power industry, and the development of more energy-efficient equipment are all highly prioritized under these policies. Hydropower, biomass, geothermal, solar, and wind power are the most common renewable energy sources, and the BRICS countries should focus on them if they want to reduce their impact on the environment.
• According to the CS-ARDL, long-term CO2 emissions are estimated to increase significantly due to industrialization. However, the AMG estimator suggests that environmental degradation may accelerate in Brazil, China, and South Africa as a result of industrialization. Modifying the industrial structures of Brazil, China, and South Africa is crucial for cutting CO2 emissions. Heavy and chemical industries, which are the main contributors to carbon dioxide (CO2) emissions, play a significant role in the BRICS countries' industrial sectors. In light of this, our policymakers should encourage zero and low-emissions sectors by stimulating industrial innovation and bolstering the reform of high-emissions industries (Liu and Bae, 2018). Renewable resources, environmentally friendly machinery, clean-burning fuel, and material recycling are all options for the industrial sectors.

## Declarations

### Author contribution statement

Liton Chandra Voumik: Conceived and designed the experiments; Performed the experiments; Analyzed and interpreted the data; Contributed reagents, materials, analysis tools or data; Wrote the paper.

Tasnim Sultana: Performed the experiments; Analyzed and interpreted the data; Wrote the paper.

### Funding statement

This research did not receive any specific grant from funding agencies in the public, commercial, or not-for-profit sectors.

### Data availability statement

Data will be made available on request.

### Declaration of interest's statement

The authors declare no conflict of interest.

### Additional information

No additional information is available for this paper.
